# Patients receiving androgen deprivation therapy for prostate cancer have an increased risk of depressive disorder

**DOI:** 10.1371/journal.pone.0173266

**Published:** 2017-03-02

**Authors:** Shiu-Dong Chung, Li-Ting Kao, Herng-Ching Lin, Sudha Xirasagar, Chung-Chien Huang, Hsin-Chien Lee

**Affiliations:** 1 Division of Urology, Department of Surgery, Far Eastern Memorial Hospital, Banciao, Taipei, Taiwan; 2 Graduate Program in Biomedical Informatics, College of Informatics, Yuan-Ze University, Chungli, Taiwan; 3 Research Center of Sleep Medicine, College of Medicine, Taipei Medical University, Taipei, Taiwan; 4 Graduate Institute of Life Science, National Defense Medical Center, Taipei, Taiwan; 5 School of Health Care Administration, Taipei Medical University, Taipei, Taiwan; 6 Department of Health Services Policy and Management, Arnold School of Public Health, University of South Carolina, Columbia, United States of America; 7 Department of Psychiatry, Taipei Medical University-Shuang-Ho Hospital, Taipei, Taiwan; 8 Department of Psychiatry, School of Medicine, College of Medicine, Taipei Medical University; University of Oklahoma Health Sciences Center, UNITED STATES

## Abstract

Androgen deprivation therapy (ADT) results in testosterone suppression, a hypothesized mechanism linking ADT to depressive symptoms. This study investigated the relationship between ADT and the risk of subsequently being diagnosed with depressive disorder (DD) during a 3-year follow-up period. The patient sample for this population-based, retrospective cohort study was retrieved from the Taiwan Longitudinal Health Insurance Database 2005. We included all 1714 patients aged over 40 years with a first-time diagnosis of prostate cancer (PC) during 2001 to 2010 who did not have an orchiectomy. Among them, we defined 868 patients who received ADT during the 3-year follow-up period as the study group, and 846 patients who did not receive ADT as the comparison group. The incidence rates of DD per 1000 person-years were 13.9 (95% confidence interval (CI): 9.5~19.6) and 6.7 (95% CI: 3.7~11.0), respectively. Cox proportional hazard regressions showed that the adjusted hazard ratio for DD for ADT recipients was 1.93 (95% CI: 1.03~3.62) relative to the comparison group. This study presents epidemiological evidence of an association between ADT and a subsequent DD diagnosis.

## Introduction

Prostate cancer (PC) is the leading cancer among men in Western countries, with about 180,000 new prostate cancer cases diagnosed in 2016 [1]. The mortality burden associated with PC is surpassed only by that of lung cancer [2]. PC remains a major health concern for older men. Hormone therapy, in the form of androgen deprivation therapy (ADT), is the standard care for newly diagnosed PC, and is increasingly accepted as an effective treatment modality for the management of prostate cancer [3]. However, ADT is known to be associated with physical and psychological side effects, such as osteopenia, sarcopenia, gynecomastia, emotional disturbances, hot flashes, fatigue, and cognitive difficulties [4–9].

ADT results in testosterone suppression, which is the mechanism hypothesized to underlie the depressive symptoms documented in some patients following ADT [10,11]. However, most studies have focused on the association between ADT and depression or depressive symptoms, rather than on a psychiatric diagnosis of depressive disorder (DD). Furthermore, studies investigating ADT and the risk of depression have shown mixed results [10–15]. Several studies by Lee, Shahinian, and Dinh reported that the rate of clinically significant depressive symptomatology was higher among PC patients treated with ADT than among those not treated with ADT [11,13,14]. In contrast, Hervoue, Timilshina, and Pirl reported that patients treated with ADT did not show any worsening of depressive symptoms [10,12,15].

To summarize the literature gaps, prior studies examined the relationship between ADT and depressive symptoms or depression, rather than a psychiatric diagnosis of DD [11,14], and the findings are equivocal regarding the association between ADT and DD. To the best of our knowledge, there is no longitudinal follow-up study of ADT and the subsequent risk of developing DD. This study used population-based data from Taiwan to investigate the risk of subsequently developing DD among ADT-treated prostate cancer patients during a 3-year follow-up period.

## Methods

### Database

The patient sample for this population-based, retrospective cohort study was retrieved from the Taiwan Longitudinal Health Insurance Database 2005 (LHID2005). The LHID2005 containts beneficiary registration files and medical claims data on one million individuals, randomly selected from all enrollees (*n* = 25.68 million) of Taiwan’s National Health Insurance (NHI) program in 2005. The LHID2005 is a de-identified dataset of all medical claims filed since 1995 for the sample individuals (1995 was the first year of NHI implementation). This dataset is available to academic researchers in Taiwan to extract samples as needed. As illustrated by several publications [16–18], hundreds of studies using this dataset have been published in international peer-reviewed journals.

Because the study dataset consists of de-identified secondary data released to the researcher community for research purposes, the study was exempted from full IRB review by the Taipei Medical University Institutional Review Board (TMU-JIRB 201612023).

### Study sample

The study was designed to include a study group and a comparison group. To select the study group, we first identified 1932 patients who received a first-time diagnosis of PC (ICD-9-CM code 185, malignant neoplasm of the prostate) at ambulatory care centers or during hospitalization, between January1, 2001 and December 31, 2010. We limited the study sample to patients aged ≥ 40 years (*n* = 1897). Patients who received ADT (gonadotropin-releasing hormone (GnRH) agonists, antiandrogens, and/or estrogens), were assigned their first ambulatory care visit with an associated ADT claim as their index date. For patients who did not receive ADT, their first claim date at which they showed a PC diagnosis became their index date. We excluded 149 patients who had an orchiectomy (ICD-9-CM procedure codes 623 or 624) during the three years following the index date. Although orchiectomy is considered equivalent to GnRH administration in its effect on testosterone levels, we excluded these patients, consistent with the study design of Dinh et al [14]. Their exclusion avoids potential confounding by depression that may occur due to the psycho-traumatic effects of the physical and anatomic changes from castration. Finally, we excluded 34 patients with pre-existing DD, those who had a diagnosis of DD (ICD-9-CM codes 296.2, 296.3, 300.4, and 311) in any claim three years prior to the index date. The final study sample consisted of 1714 PC patients, 868 patients who received ADT, the study group, and 846 patients who did not receive ADT, the comparison group. These selection criteria are consistent with prior studies [16].

We tracked each sampled patient’s claims over a 3-year period following their index date to identify those who subsequently received a diagnosis of a DD. The diagnosis of DD was defined as the presence of depressive disorder codes in inpatient claims data. The ICD-9-CM codes used to identify the end points in this study have not been formally validated but have been used in previous studies [18–20].

### Statistical analysis

We conducted statistical analyses using the SAS statistical software (SAS System for Windows, V.9.2, SAS Institute). We used chi-squared tests to explore differences between the study group and comparison group on sociodemographic characteristics (monthly income, residential urbanization level and geographic location) and medical comorbidities (hypertension, diabetes, hyperlipidemia, coronary heart disease (CHD), and stroke). We performed Cox proportional hazard regressions to calculate the adjusted hazard ratio (HR) for DD and the 95% confidence interval (CI) over 3-year follow-up. We verified that the proportional hazards assumption was satisfied, based on survival curves for both strata (study group and comparison group) showing hazard functions that were proportional over time. Patients who died during follow-up were censored. We used a two-sided *p* value of 0.05 for statistical significance.

## Results

Table 1 presents the sample distribution by demographic characteristics and medical comorbidities of patients classified by ADT status. PC patients who received ADT were on average, older than the comparison group (74.1 vs. 70.4 years, *p*<0.001), and more likely to reside in the southern part of Taiwan (*p*<0.001), and to have lower monthly income (<NT$15,841 (*p*<0.001) compared to those who did not receive ADT. There was no significant difference in the prevalence of comorbidities, hypertension, diabetes, hyperlipidemia, or CHD.

**Table 1:** Demographic characteristics of patients with prostate cancer classified by receipt of Androgen Deprivation Therapy (ADT) (*N* = 1714).

Variable	Patients who received ADT (*n* = 868)		Patients who did not receive ADT (*n* = 846)		*p* value
	Total no.	Column %	Total no.	Column %	
Age (years), mean (SD)	74.1 (8.4)		70.4 (10.8)		<0.001
Urbanization level					0.178
1 (most urbanized)	269	31.0	276	32.6	
2	236	27.2	261	30.8	
3	133	15.3	111	13.1	
4	121	13.9	113	13.4	
5 (least urbanized)	109	12.6	85	10.1	
Geographic region					<0.001
Northern	406	46.8	490	57.9	
Central	194	22.3	177	20.9	
Southern	247	28.5	166	19.6	
Eastern	21	2.4	13	1.5	
Monthly income					<0.001
NT$0~15,840	554	63.8	495	58.5	
NT$15,841~25,000	239	27.5	205	24.2	
≥NT$25,001	75	8.6	146	17.3	
Hypertension	618	71.2	568	67.1	0.069
Diabetes	249	28.7	237	28.0	0.757
Hyperlipidemia	334	38.5	337	39.8	0.566
Coronary heart disease	320	36.9	312	36.9	0.996
Stroke	285	32.8	239	28.3	0.040

*Note*: The average exchange rate in 2015 was US$1.00≈New Taiwan (NT)$32.

Table 2 shows the incidence of DD among sample patients at 10.3 per 1000 person-years (95% CI: 7.6~13.7). The rates were13.9 (95% CI: 9.5~19.6) and 6.7 per 1000 person-years (95% CI: 3.7~11.0), respectively, among the study and comparison groups. A total of 47 patients (2.74%) of the total sample patients received a DD diagnosis after the index date, 32 patients (3.69%) among the study group and 15 patients (1.77%) among the comparison group. The log-rank test revealed that study patients were significantly more likely to receive a DD diagnosis than the comparison group (*p* = 0.012). The unadjusted HR for DD among the study group was 2.12 (95% CI: 1.14~3.95) relative to the comparison group. We also compared the total sample patients who received a DD diagnosis during follow-up with the number of PC patients who had a DD diagnosis prior to their PC diagnosis (these patients were excluded from the study due to pre-existing DD, 34 patients). The finding is that the number of patients with a post-PC diagnosis of DD (47 patients) exceeds the number with preexisting DD.

**Table 2:** Crude Hazard Ratios (HRs) for depressive disorder among patients with prostate cancer during a 3-year follow-up period, stratified by receipt of Androgen Deprivation Therapy (ADT).

Presence of depressive disorder	Total sample (*n* = 1714)	Patients who received ADT (*n* = 868)	Patients who did not receive ADT (*n* = 846)
Three-year follow-up period			
Incidence rate per 1000 person-years (95% CI)	10.3 (7.6~13.7)	13.9 (9.5~19.6)	6.7 (3.7~11.0)
Crude HR (95% CI)	--	2.12* (1.14~3.95)	1.00

*Notes*:

* *p*≤0.05;

CI, confidence interval.

Table 3 presents the adjusted HR for study group patients over 3-year follow-up relative to the comparison group. The HR for DD was 1.93 (95% CI: 1.03~3.62) after adjusting for geographic location, monthly income, urbanization level, and comorbidities. It is noteworthy that stroke was significantly associated with DD risk (adjusted HR = 2.09; 95% CI: 1.14~3.86). Geographical location, monthly income, urbanization level, hypertension, diabetes, hyperlipidemia, and CHD were not associated with DD.

**Table 3:** Adjusted hazard ratio for the occurrence of depressive disorder during 3-year follow-up among patients with prostate cancer (*n* = 1714).

Variable	Depressive disorder occurrence		
	Hazard ratio	95% CI	*p* value
ADT recipients	1.93	1.03~3.62	0.041
Geographic region			
Northern	1.00		
Central	1.31	0.60~2.88	0.501
Southern	0.96	0.43~2.17	0.924
Eastern	2.73	10.95	0.157
Monthly income			
NT$0~15,840	1.00		
NT$15,841~25,000	0.85	0.41~1.77	0.665
≥NT$25,001	0.44	0.10~1.90	0.274
Urbanization level			
1 (most urbanized)	1.00		
2	0.59	0.23~1.54	0.282
3	1.14	0.42~3.12	0.796
4	2.16	0.83~5.65	0.114
5 (least urbanized)	1.71	0.58~5.03	0.327
Hypertension	2.05	0.92~4.57	0.081
Diabetes	1.13	0.58~2.20	0.717
Hyperlipidemia	0.89	0.47~1.70	0.721
Coronary heart disease	1.18	0.64~2.18	0.605
Stroke	2.09	1.14~3.86	0.018

*Notes*: All hazard ratios are reported from a single Cox regression model adjusted for all other variables.

The average exchange rate in 2015 was US$1.00≈New Taiwan (NT)$32.

CI, confidence interval.

## Discussion

This is the first population-based study to explore the longitudinal association between ADT and subsequent development of DD over 3-year follow-up in an Asian country. We find that patients treated with ADT have double the DD incidence found among those not treated with ADT (13.9 vs. 6.7 per 1000 person-years). The unadjusted HR was 2.12, and after adjusting for demographic and co-morbidity characteristics, the HR was 1.93.To date, the association between ADT and depression has been equivocal. Two studies by Hervouet and Timilshina using a small study sample failed to show an association between ADT and the development of depressive symptoms [10,15]. By comparison, other studies are consistent with our research findings. Shahinian and Dinh, using linked SEER-Medicare data, found that the relative risk for a depression diagnosis among those with ADT was 1.08, and the HR was 1.23, relative to those without ADT [13,14]. Dinh et al. reported that patients who received ADT had higher 3-year cumulative incidence rates of depression (7.1% vs. 5.2%), outpatient psychiatric treatment (3.4% vs. 2.5%), and inpatient psychiatric treatment (2.8% vs. 1.9%), than those who did not receive ADT [14]. The magnitude of difference between the two groups is consistent with our study (3.69% cumulative incidence of DD over 3 years among ADT recipients vs. 1.77% among the comparison group). A literature review by Donovan et al. documented that ADT is associated with adverse psychological effects, including difficulties in multiple indicators in the sexual domains, emotional lability, depression, and cognitive impairment [21]. One study using a structured questionnaire from Melbourne reported that patients on long-term ADT had poor quality of life and psychosocial well-being [22]. These studies support an association between ADT and DD.

ADT results in testosterone suppression, the hypothesized mechanism linking ADT to depressive symptoms [10,11]. Several studies have shown an association between the progressive decline in testosterone levels and increasing risk of depressive symptoms [10,11,14,23–25]. Seidman and colleagues hypothesized that lower testosterone levels may be etiologically important in the development of depressive syndromes in elderly men, and found that a majority of elderly men with dysthymic disorder had total testosterone levels in the hypogonadal range (i.e., ≤300 ng/dl) [26]. The evidence supports that declines in testosterone levels may play a role in the development of DD. The drastic testosterone depletion induced by ADT, plausibly, could lead to DD in PC patients.

Another mediating mechanism may be the physiological changes resulting from ADT [4,6,11]. Gray and colleagues noted that most men with PC avoided disclosing their illness and tended to conceal their emotional difficulties or deny the support they needed [27]. In addition, some studies suggested that psychological distress resulting from initiation of ADT might increase the depressive symptomatology of patients receiving ADT [4,6]. Consequently, physiological changes, interacting with the affective response to the PC diagnosis and ADT effects, may induce the development of DD.

Other mechanisms are also postulated including ADT-induced changes in the immune system. Aragon-Ching and colleagues suggest that ADT impacts the immune system [28]. A connection between depression and immune parameters, such as, pro-inflammatory cytokines (e.g., interleukin (IL)-1 and IL-6) has been explored [29]. ADT-induced changes in immunity may influence the development of DD in patients with PC. More research is needed to explore our finding and the mechanisms underlying this association.

Although our primary focus was to explore the association of ADT with DD, the risk of cardiovascular events is also important among ADT recipients. One cross-sectional study in Italy concluded that inappropriate ADT was associated with increased risks of cardiovascular disease and osteoporosis [30]. A meta-analysis reported that patients with intermittent ADT had a lower risk of cardiovascular-related mortality [31]. Further study is needed to evaluate the relationship between ADT and cardiovascular events, and a potential interaction with the risk of DD.

Lack of data on cancer stage is an important study limitation. It is possible that cancer stage is a confounding factor in our findings. The likelihood of finding a DD may be higher among early-stage cancer patients, simply because they are less likely to die before the end of follow-up. Mitigating this bias is the fact that GnRH group is likely to have more advanced cancer cases, and therefore, a stronger, disease-driven reason for starting hormone therapy. The mean age of the ADT group was significantly higher. Patients with more advanced cancers may be more prone to depression, and they also are more likely to be started on GnRH treatment, which would imply an upward bias in depression HR estimates among this group relative to the non-ADT group. But, older age at PC diagnosis would also be associated with more deaths during the observation period. Censoring patients at death and controlling for age in the hazard function modelling, corrects for such bias to some extent. Nevertheless, confounding by differences in cancer stage between the study and control groups cannot be ruled out.

Other limitations should also be noted. First, clinician bias in diagnosing DD among ADT patients cannot be ruled out, driven a heightened awareness of DD predisposing factors, the patient’s age, comorbidities, advanced cancer, and complaints of the physical effects of ADT. Adjusting for age, geographical location, monthly income, urbanization level, hypertension, diabetes, hyperlipidemia, CHD, and stroke in the regression model may have attenuated this source of bias. We find a consistent, increased risk of subsequent DD during 3-year follow-up among PC patients receiving ADT. Another potential study limitation is surveillance bias, due to PC patients on ADT being clinically monitored more frequently than those on ADT, causing physicians to be more likely to detect DD symptoms among these patients. Finally, another limitation is the lack of data on PC patients' medication adherence.

The strength of our study lies in its longitudinal design and large population-based sample, which mitigates selection bias that is inherent in studies utilizing data from voluntary registries or hospital-based patients. Furthermore, given that over 98% of Taiwan’s residents are of Chinese Han ethnicity, use of this homogenous population eliminates confounding by race.

Despite the limitations, our study offers strong epidemiological evidence of a link between ADT and a subsequent DD diagnosis. The study suggests that PC patients receiving ADT may benefit from screening for the modifiable risk factors for depressive disorder, and also periodic assessments for depressive symptoms. Patients should also be informed about this possible adverse effect when discussing treatment options among PC patients.

## References

[1] Howlader N, Noone AM, Krapcho M, Miller D, Bishop K, Altekruse SF, et al SEER cancer statistics review, 1975–2013. National Cancer Institute; Bethesda, MD: 2016 Released April 15, 2016. http://seer.cancer.gov/csr/1975_2013/results_single/sect_01_table.01.pdf [accessed May 1, 2016].

[2] Cooperberg MR, Broering JM, Litwin MS, Lubeck DP, Mehta SS, Henning JM, et al The contemporary management of prostate cancer in the United States: lessons from the cancer of the prostate strategic urologic research endeavor (CapSURE), a national disease registry. J Urol. 2004;171:1393–1401. 10.1097/01.ju.0000107247.81471.06 15017184

[3] Labrie F. Medical castration with LHRH agonists: on survival in prostate cancer. J Androl. 2004;25: 305–313. 15064303 10.1002/j.1939-4640.2004.tb02791.x

[4] Chen AC, Petrylak DP. Complications of androgen deprivation therapy in men with prostate cancer. Curr Oncol Rep. 2004;6: 209–215. 15066232 10.1007/s11912-004-0051-0

[5] Taylor LG, Canfield SE, Du XL. Review of major adverse effects of androgen deprivation therapy in men with prostate cancer. Cancer. 2009;115: 2388–2399. 10.1002/cncr.24283 19399748

[6] Casey RG, Corcoran NM, Goldenberg SL. Quality of life issues in men undergoing androgen deprivation therapy: a review. Asian J Androl. 2012;14: 226–231. 10.1038/aja.2011.108 22231296 PMC3735091

[7] Seidman SN, Walsh BT. Testosterone and depression in aging men. Am J Geriatr Psychiatry. 1999;7: 18–33. 9919317

[8] Shores MM Sloan KL, Matsumoto AM, Moceri VM, Felker B, Kivlahan DR. Increased of diagnosed illness in hypogonadal older men. Arch Gen Psychiatry. 2004;61: 162–167. 10.1001/archpsyc.61.2.162 14757592

[9] Nguyen PL, Alibhai SM, Basaria S, D'Amico AV, Kantoff PW, Keating NL, et al Adverse effects of androgen deprivation therapy and strategies to mitigate them. Eur Urol. 2015;67: 825–836. 10.1016/j.eururo.2014.07.010 25097095

[10] Hervouet S, Savard J, Ivers H, Savard MH. Depression and Androgen deprivation therapy for Prostate cancer: a prospective controlled study. Health Psychol. 2013;32: 675–684. 10.1037/a0031639 23477572

[11] Lee M, Jim HS, Fishman M, Zachariah B, Heysek R, Biagioli M, et al Depressive symptomatology in men receiving Androgen deprivation therapy for Prostate cancer: a controlled comparison. Psychooncology. 2015;24, 472–477. 10.1002/pon.3608 24924331 PMC4265307

[12] Pirl WF, Siegel GI, Goode MJ, Smith MR. Depression in men receiving Androgen deprivation therapy for Prostate cancer: a pilot study. Psychooncology 2002;11: 518–523. 10.1002/pon.592 12476433

[13] See comment in PubMed Commons below Shahinian VB, Kuo YF, Freeman JL, Goodwin JS. Risk of the "androgen deprivation syndrome" in men receiving androgen deprivation for Prostate cancer. Arch Intern Med. 2006;166: 465–471. 16505268 10.1001/.465 PMC2222554

[14] Dinh KT, Reznor G, Muralidhar V, Mahal BA, Nezolosky MD, Choueiri TK, et al Association of Androgen deprivation therapy With Depression in Localized Prostate cancer. J Clin Oncol. 2016;34: 1905–1912 10.1200/JCO.2015.64.1969 27069075 PMC4966343

[15] Timilshina N, Breunis H, Alibhai S. Impact of androgen deprivation therapy on depressive symptoms in men with nonmetastatic prostate cancer. Cancer. 2012;118: 1940–1945. 10.1002/cncr.26477 22009684

[16] Wang LH, Liu CK, Chen CH, Kao LT, Lin HC, Huang CY. No increased risk of coronary heart disease for patients receiving androgen deprivation therapy for prostate cancer in Chinese/Taiwanese men. Andrology. 2016;4: 128–132. 10.1111/andr.12141 26711703

[17] Chen HM, Lin CC, Kang CS, Lee CT, Lin HC, Chung SD. Bladder pain syndrome/interstitial cystitis increase the risk of coronary heart disease. Neurourol Urodyn. 2014;33: 511–515. 10.1002/nau.22444 23813657

[18] Huang CY, Chiu KM, Chung SD, Keller JJ, Huang CC, Lin HC. Increased risk of depressive disorder following the diagnosis of benign prostatic enlargement: one-year follow-up study. J Affect Disord. 2011; 135: 395–399. 10.1016/j.jad.2011.07.001 21824662

[19] Tsai MC, Chen CH, Lee HC, Lin HC, Lee CZ. Increased Risk of Depressive Disorder following Cholecystectomy for Gallstones. PLoS One. 2015;10: e0129962 10.1371/journal.pone.0129962 26053886 PMC4460135

[20] Chung SD, Ho JD, Hwa P, Lee HC, Lin HC. Increased risk of depressive disorder following a diagnosis of neovascular age-related macular degeneration. Acta Ophthalmol. 2015;93: e176–177. 10.1111/aos.12478 25588712

[21] Donovan KA, Walker LM, Wassersug RJ, Thompson LM, Robinson JW. Psychological effects of androgen-deprivation therapy on men with prostate cancer and their partners. Cancer. 2015;121: 4286–4299. 10.1002/cncr.29672 26372364

[22] Chipperfield K, Fletcher J, Millar J, Brooker J, Smith R, Frydenberg M, et al Predictors of depression, anxiety and quality of life in patients with prostate cancer receiving androgen deprivation therapy. Psychooncology. 2013;22: 2169–2176. 10.1002/pon.3269 23483679

[23] Barrett-Connor E, Von Mühlen DG, Kritz-Silverstein D. Bioavailable testosterone and depressed mood in older men: the Rancho Bernardo Study. J Clin Endocrinol Metab. 1999;84: 573–577. 10.1210/jcem.84.2.5495 10022418

[24] McIntyre RS, Mancini D, Eisfeld BS, Soczynska JK, Grupp L, Konarski JZ, et al Calculated bioavailable testosterone levels and depression in middle-aged men. Psychoneuroendocrinology. 2006;31: 1029–1035. 10.1016/j.psyneuen.2006.06.005 16908107

[25] Shores MM, Moceri VM, Sloan KL, Matsumoto AM, Kivlahan DR. Low testosterone levels predict incident depressive illness in older men: effects of age and medical morbidity. J Clin Psychiatry.2005; 66:7–14. 10.4088/jcp.v66n0102 15669883

[26] Seidman SN, Araujo AB, Roose SP, Devanand DP, Xie S, Cooper TB, et al Low testosterone levels in elderly men with dysthymic disorder. Am J Psychiatry. 2002;159: 456–459. 10.1176/appi.ajp.159.3.456 11870011

[27] Gray RE, Fitch M, Phillips C, Labrecque M, Fergus K. To tell or not to tell: patterns of disclosure among men with prostate cancer. Psychooncology. 2000;9: 273–282. 10960925 10.1002/1099-1611(200007/08)9:4<273::aid-pon463>3.0.co;2-f

[28] Aragon-Ching JB, Williams KM, Gulley JL. Impact of androgen-deprivation therapy on the immune system: implications for combination therapy of prostate cancer. Front Biosci. 2007;12: 4957–4971. 17569623 10.2741/2441

[29] Vedhara K, Irwin M. Human psychoneuroimmunology. New York, NY: Oxford University Press; 2005.

[30] Morgia G, Russo GI, Tubaro A, Bortolus R, Randone D, Gabriele P, et al Prevalence of Cardiovascular Disease and Osteoporosis During Androgen Deprivation Therapy Prescription Discordant to EAU Guidelines: Results From a Multicenter, Cross-sectional Analysis From the CHOsIng Treatment for Prostate canCEr (CHOICE) Study. Urology. 2016;96: 165–170. 10.1016/j.urology.2016.06.024 27402374

[31] Jin C, Fan Y, Meng Y, Shen C, Wang Y, Hu S, et al A meta-analysis of cardiovascular events in intermittent androgen-deprivation therapy versus continuous androgen-deprivation therapy for prostate cancer patients. Prostate Cancer Prostatic Dis. 2016;19: 333–339. 10.1038/pcan.2016.35 27595915

